# Effect of silibinin on endothelial dysfunction and ADMA levels in obese diabetic mice

**DOI:** 10.1186/1475-2840-10-62

**Published:** 2011-07-14

**Authors:** Giovanni Li Volti, Salvatore Salomone, Valeria Sorrenti, Andrea Mangiameli, Vincenzo Urso, Ilias Siarkos, Fabio Galvano, Federico Salamone

**Affiliations:** 1 Department of Drug Sciences, University of Catania, Catania, Italy; 2 Department of Cardiac Surgery, San Donato Institute, San Donato Milanese (MI), Italy; 3 Department of Clinical and Molecular Biomedicine, University of Catania, Catania, Italy; 4 Department of Internal Medicine, University of Catania, Catania, Italy

**Keywords:** diabetes, silibinin, db/db, endothelial dysfunction, ADMA, vascular reactivity

## Abstract

**Background:**

Cardiovascular diseases (CVD) in diabetic patients have endothelial dysfunction as a key pathogenetic event. Asymmetric dimethylarginine (ADMA), an endogenous inhibitor of nitric oxide synthase (NOS), plays a pivotal role in endothelial dysfunction. Different natural polyphenols have been shown to preserve endothelial function and prevent CVD. In this study, we assessed the effect of silibinin, a widely used flavonolignan from milk thistle, on ADMA levels and endothelial dysfunction in db/db mice.

**Methods:**

Eight-week-old db/db mice were administrated a 20 mg/Kg i.p. daily dose of silibinin (n = 6) or vehicle (n = 6) for four weeks. Heterozygous lean db/m mice served as control. Plasma, aorta and liver ADMA levels were determined by ELISA. Vascular reactivity to phenilephrine (PE), acetylcholine (ACh), sodium nitroprusside (SNP) and ADMA was assessed in isolated aortic segments, in wire myograph.

**Results:**

Plasma and aorta ADMA levels were higher in db/db than in control lean mice. Silibinin administration markedly decreased plasma ADMA; consistently, aorta ADMA was reduced in silibinin-treated animals. Plasma and aorta ADMA levels exhibited a positive correlation, whereas liver ADMA was inversely correlated with both plasma and aorta ADMA concentrations. Endothelium-(NO)-dependent vasodilatation to ACh was impaired in db/db mice and was restored in the silibinin group, in accordance with the observed reduction of plasma and vascular levels of ADMA. Endothelium-independent vasodilatation to SNP was not modified by silibinin administration; contractile tone induced in isolated aorta from db/db mice by challenging with exogenous ADMA was not affected by the treatment.

**Conclusions:**

Silibinin markedly improves endothelial dysfunction in db/db mice by reducing circulating and vascular ADMA levels. Clinical studies are warranted to assess the efficacy of silibinin for cardiovascular protection.

## Background

Cardiovascular diseases (CVD) are a leading cause of death worldwide and are tightly related to obesity, diabetes and the full spectrum of the metabolic syndrome [1]. Endothelial dysfunction is a main event in the pathogenetic cascade conducing to cardiovascular events [2] and therapies aiming at preserving endothelium are needed for the effective prevention of CVD. Endothelium plays a central role in the physiological maintenance of vascular function by regulating vascular tone, leukocyte adhesion and platelet activation, as a result of release of vasoactive substances such as nitric oxide (NO), prostacyclin and endothelin [3].

Several natural polyphenols have been evaluated for their ability to preserve endothelial function and thus for their effectiveness in preventing CVD [4]. Recently, silibinin, a polyphenolic compound contained in silymarin, has been demonstrated to exert *in vitro *protective effects on endothelial cells [5]; however, it is not clear whether silibinin may exert vascular protective effect *in vivo*.

In the diabetic status, vascular dysfunction is mainly related to decreased NO bioavailability [6]. Previous studies showed that endogenous arginine analogs may play a regulatory role in the arginine/NO pathway [7]. Asymmetric N^G^, N^G^-dimethyl-L-arginine (ADMA) is an endogenous inhibitor of all isoforms of Nitric Oxide Synthase (NOS). Several evidences suggest that ADMA exerts deleterious vascular effects by inhibiting endothelial NOS (eNOS). Elevated ADMA levels have been identified as a biomarker of endothelial dysfunction [8]. Interestingly, circulating ADMA is significantly correlated with carotid artery intima-media thickness [9], and ADMA increase precedes the occurrence of vascular occlusive disease [10]. Taken together, these observations suggest that plasma ADMA is significantly associated with cardiovascular risk. ADMA metabolism is related to its generation from protein breakdown and to its cleavage by dimethylarginine hydrolase (DDAH) into citrulline and dimethylamine [11].

The liver is a main regulator of circulating ADMA levels [12]. Nijvedt documented a daily hepatic ADMA extraction for more than seven hundred times the amount of plasma ADMA in rats [13]. Serum ADMA is increased in patients with diabetes [14] and in non-diabetic insulin-resistant subjects [15,16], and improvement in insulin resistance is associated to a decrease of ADMA [17,18]. Animal models of diabetes, such as db/db mice, are widely studied because they reproduce several metabolic and vascular alterations occurring in humans; endothelial dysfunction has been documented in db/db mice [19-24], however, to the best of our knowledge, the association of ADMA levels with endothelial dysfunction has not been investigated in this animal model. In this study we assessed plasma, aorta and liver ADMA levels and analyzed endothelium-dependent vascular reactivity in db/db mice; furthermore, we examined the effects of silibinin administration on these parameters.

## Methods

### Animals and treatments

All procedures were carried out in accordance with the Italian Guidelines for the Care and Use of Laboratory Animals and with the ARRIVE guidelines [25]. Eight-week-old male BKS.Cg-m+/+ Leprdb/J (db/db) obese mice and eight-week-old male heterozygous db/m lean control mice were purchased from Charles River Lab (Calco, Italy). Animals were housed at constant room temperature (23°C) (n = 3 per cage) under 12 hours light/dark cycles with *ad libitum *access to water and were fed a standard diet. Silibinin dihydrogen disuccinate disodium (Madaus, Köln, Germany) was dissolved in saline which was also used as vehicle for the placebo-treated animals. Eighteen mice were distributed in 3 groups: group I (db/m + vehicle) included six heterozygous db/m mice treated with saline; group II (db/db + vehicle) comprised six db/db mice treated with saline; group III (db/db + silibinin) included six db/db mice treated with silibinin (20 mg/Kg i.p. injection, daily). At 12 weeks of age, after an overnight fast, animals were anesthetized and sacrificed; blood, aorta and liver samples were obtained for further analysis.

### Biochemical analyses

Blood glucose was measured by Accu-chek (Roche Diagnostics, Milan, Italy). Serum insulin was determined using an automated enzyme immunoassay analyzer from Tosoh (Tokio, Japan). HOMA-IR was calculated as follows: [fasting glucose (mmol/L) × fasting insulin (mU/L)]/22.5. Plasma and tissue ADMA concentration was determined by using a commercially available enzyme-linked immunosorbent assay kit (DLD Diagnostika GmbH, Hamburg, Germany) according to the manufacturer's instructions.

### Vasomotor tone

Aortas were immersed in physiological salt solution (composition, mmol/L: NaCl, 118; KCl, 4.6; NaHCO_3_, 25; MgSO_4_, 1.2; KH_2_PO_4_, 1.2; CaCl_2_, 1.2; glucose, 10; EDTA, 0.025; pH 7.4 at 37°C), dissected out of surrounding connective tissue, cut in segments (2 mm length) and mounted in a wire myograph (610 M, Danish Myo Technology, Aarhus, Denmark), by using 40 μm-diameter stainless steel wire, for isometric record of contractile force. After mounting, each preparation was equilibrated unstretched, for 30 min, in physiologic salt solution, maintained at 37°C and aerated with a gas mixture 95% O_2 _- 5% CO_2_. The normalized passive resting force and the corresponding diameter were then determined for each preparation from its own length-pressure curve, according to Mulvany and Halpern [26]. Contractile responses were recorded into a computer, by using a data acquisition and recording software (Myodaq and Myodata, Danish Myo Technology). After normalization and 30-min equilibration in physiological solution, the preparations were constricted with isotonic depolarizing KCl solutions, in which part of NaCl had been replaced by an equimolar amount of KCl (composition, mmol/L: NaCl, 22.6; KCl, 98.8; NaHCO_3_, 25; MgSO_4_, 1.2; KH_2_PO_4_, 1.2; CaCl_2_, 1.2; glucose, 10; EDTA, 0.025, pH 7.4 at 37°C). After washout and 30-min recovery, the preparations were exposed to cumulative concentrations of phenilephrine (PE, 1 nmol/L - 1 μmol/L). Once the vasoconstriction to PE had reached steady state, cumulative concentrations of acetylcholine (ACh, 1 nmol/L - 1 μmol/L) were added to the organ chamber. Relaxing responses were expressed in percentage of pre-existing contractile tone, induced by PE. After wash out and 30 min recovery, preparations were exposed to 5 nmol/L PE, which induced a negligible (< 2% of K^+^100) vasoconstriction in lean, but a stronger one in db/db (17-18% of K^+^100); once PE-induced tone had reached a steady state, preparations were challenged with cumulative concentrations of asymmetric N^G^, N^G^-dimethyl-L-arginine (ADMA, 300 nmol/L - 300 μmol/L). Preparations were then further constricted by 1 μmol/L PE in the presence of 100 μmol/L N^G^-nitro-L-arginine (LNNA), once PE-induced tone had reached a steady state, preparations were challenged with cumulative concentrations of sodium nitroprusside (SNP, 1 nmol/1 L - 1 μmol/L), to assess endothelium-independent vasodilatation. PE, ACh, SNP, LNNA and ADMA were from Sigma (St. Louis, MO, U.S.A.); 10 mmol/L stock solutions were prepared in water and further diluted in physiological salt solution as required, except for ADMA, which was dissolved in water as 30 mmol/L stock solution. Concentration-response curves to vasoactive agonists were plotted as response (for vasoconstrictors, in % of vasoconstriction induced by KCl in the same arteries; for vasodilators in % of residual tone) against log molar concentration of drug. Each set of data points was curve-fitted by a non-linear regression, best-fit, sigmoid dose-response curve with no constraints, with the use of Graph Pad Prism (Graph Pad Software, San Diego, CA). Pharmacological parameters (concentration producing 50% of maximum effect or EC_50 _and maximum effect or E_max_) were calculated from these non-linear fits. Each curve represents 12 preparations from 6 different mice.

### Statistical analysis

Statistics were aided by Graph Pad Prism. All results were expressed as mean ± standard error of mean (S.E.M.). P values less than 0.05 were considered significant. Biochemical data were analyzed by one-way ANOVA with Bonferroni Post-Hoc analysis. Concentration-response curve from isolated aortas were compared by two-way ANOVA.

## Results

### Metabolic parameters

All db/db mice weighted more than lean controls at week 8, before starting the treatment; silibinin administration did not significantly change body weight or food intake (data not shown). Untreated db/db were insulin resistant, as shown by HOMA-IR; silibinin decreased fasting glucose and insulin, reversing insulin resistance (Table 1).

**Table 1:** Biometric and biochemical parameters

	db/m + vehicle	db/db + vehicle	db/db + silibinin
**8-weeks weight** **(g)**	22.5 ± 0.5	33.0 ± 2.2*	32.5 ± 2.7*

**12-weeks weight** **(g)**	25.6 ± 1.2	38.4 ± 1.8*	36.8 ± 1.9*

**Blood glucose (mg/dL)**	68.2 ± 7.6	220.8 ± 40.2**	112.7 ± 22.8^†^

**Serum insulin (mU/L)**	6.8 ± 1.2	10.3 ± 0.8**	7.5 ± 1.1^†^

**HOMA-IR**	1.1 ± 0.2	5.6 ± 0.5**	2.1 ± 0.3^††^

*P < 0.05, **P < 0.01 versus db/m + vehicle, ^†^P < 0.05, ^†† ^P < 0.01 versus db/db + vehicle.

### Plasma, aorta and liver ADMA levels

**Figure 1:**
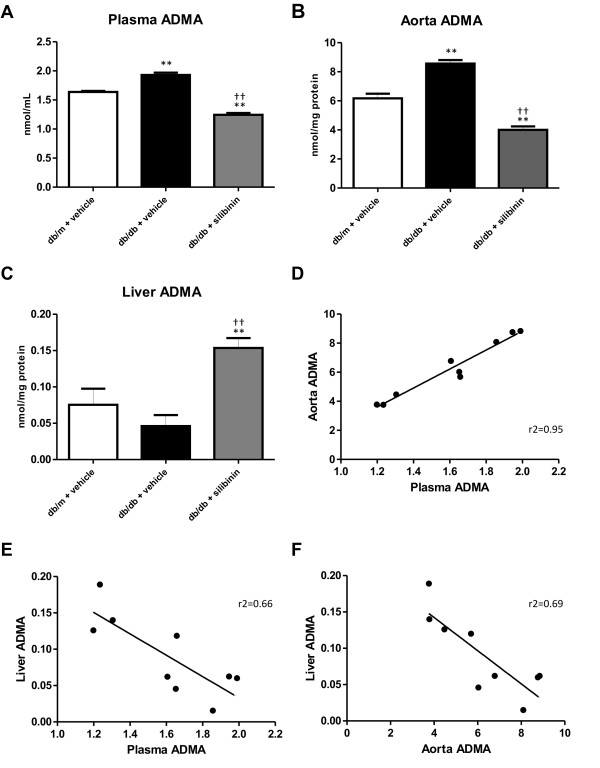
**Plasma, aorta and liver ADMA levels.** (A) Plasma ADMA was higher in db/db mice than lean controls and was significantly decreased by silibinin administration. (B) Aorta ADMA was similarly higher in untreated db/db mice and was markedly decreased in mice treated with silibinin. (C) Liver ADMA was increased in the silibinin group. (D) Plasma and aorta ADMA levels exhibited a positive correlation (r2=0.95, P<0.0001). (E, F) Liver ADMA levels were inversely related to both plasma ADMA (r2=0.66, P<0.01) and aorta ADMA (r2=0.69, P<0.01). **P < 0.01 versus db/m + vehicle; ††P < 0.01 versus db/db + vehicle.

Plasma ADMA levels were higher in db/db than in db/m (Figure 1A). Silibinin reduced plasma ADMA in db/db to a level lower than in db/m animals. These differences in ADMA levels were even more pronounced in aorta, where, again, ADMA was higher in db/db compared to db/m and was reduced by silibinin in db/db mice, by more than 50% (Figure 1B). Liver ADMA was slightly lower in db/db as compared to lean controls, although the difference was not statistically significant, whereas silibinin led to raised ADMA in hepatic tissue (Figure 1C). Plasma and aorta ADMA levels were positively correlated (Figure 1D); by contrast, liver ADMA was inversely correlated with both plasma and aorta ADMA concentrations (Figure 1E).

### Vascular tone

Contractile tone induced by high K^+ ^was higher in aorta preparations from db/db than in db/m mice and was not influenced by silibinin treatment (db/m + vehicle, 3.42 ± 0.25 mN/mm; db/db + vehicle, 4.33 ± 0.35 mN/mm; db/db + silibinin, 4.36 ± 0.27 mN/mm; db/m + vehicle versus all db/db, P < 0.05).

**Figure 2:**
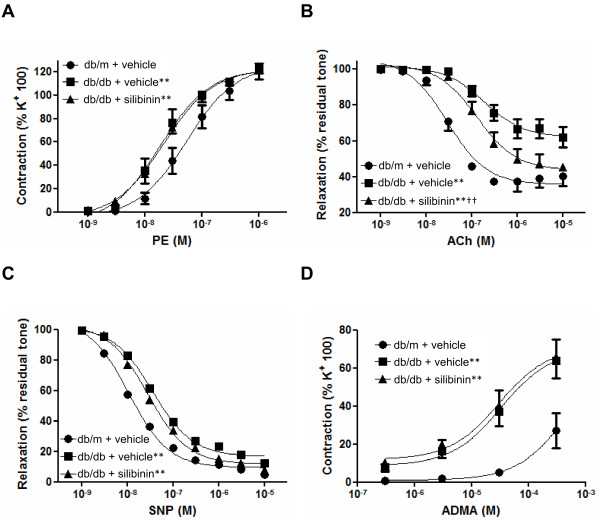
**Vasomotor responses in isolated aorta**. (A) Vasoconstriction to phenilephrine (PE); (B) vasodilatation to acetylcholine (ACh); (C) vasodilatation to sodium nitroprusside (SNP); (D) vasoconstriction to asymmetric N^G^, N^G^-dimethyl-L-arginine (ADMA). Vasoconstriction is expressed in % of high K^+^-induced tone in the same preparation; vasodilatation is expressed as residual tone in % of PE-induced preconstriction. **P < 0.01 versus db/m + vehicle; ^††^P < 0.01 versus db/db + vehicle.

Vasoconstriction to PE, either expressed as raw values (mN/mm, not shown) or normalized in % of K^+^-induced contraction (Figure 2A and Table 2) was also higher in db/db; in particular concentration-contraction curve to PE was displaced to the left in db/db mice, in a parallel manner. Vasoconstriction to PE was not modified by silibinin administration. Endothelium-dependent vasodilatation to ACh and, to a less extent, endothelium-independent vasodilatation to SNP were lower in db/db mice as compared to lean animals (Figure 2B, C and Table 2); vasodilatation to ACh was significantly increased by silibinin, whereas endothelium-independent vasodilatation to SNP was not modified by the treatment. *In vitro *challenge with ADMA induced a stronger contractile tone in aorta from db/db mice than in db/m (Figure 2D); of note, the tone induced by applying ADMA *in vitro *in aorta from db/db mice, was not influenced by *in vivo *silibinin.

**Table 2:** Pharmacological parameters of vasomotor responses to phenilephrine **(**PE), acetylcholine **(**Ach), sodium nitroprusside **(**SNP) and asymmetric N^G^, N^G^-dimethyl-L-arginine **(**ADMA) in isolated aorta

PE			
	pD_2_	EC_50 _(nmol/L)	E_max _(% K^+^)
db/m + vehicle	7.27 ± 0.11	53.7 (32.1-89.7)	126.0 ± 7.0
db/db + vehicle	7.74 ± 0.10**	18.3 (11.3-29.9)	122.3 ± 5.0
db/db + silibinin	7.66 ± 0.08**	21.7 (15.3-30.7)	122.6 ± 3.7

**ADMA**			
	**pD_2_**	**EC_50 _(**μ**mol/L**)	**E_max _(% K^+^)**

db/m + vehicle	3.36 ± 1.76	440 (0.1-10^6^)	65.6 (± 154)
db/db + vehicle	4.48 ± 0.36**	32.8 (5.8-188)	69.6 ± 12.0
db/db + silibinin	4.51 ± 0.40**	30.1 (4.8-194)	71.2 ± 12.6

**ACh**			
	**pD_2_**	**EC_50 _(nmol/L)**	**E_max_(% tone)**

db/m + vehicle	7.54 ± 0.12	28.8 (16.7-49.5)	35.7 ± 2.5
db/db + vehicle	6.74 ± 0.15**	183 (90.2-372)	62.1 ± 2.1**
db/db + silibinin	6.89 ± 0.12**	130 (74.9-224)	43.7 ± 2.8^††^

**SNP**			
	**pD_2_**	**EC_50 _(nmol/L)**	**E_max_(% tone)**

db/m + vehicle	7.97 ± 0.04	10.1 (8.8-12.9)	9.4 ± 1.0
db/db + vehicle	7.45 ± 0.06**	35.7 (26.5-48.0)	16.7 ± 1.7*
db/db + silibinin	7.56 ± 0.05**	27.7 (21.7-35.3)	11.8 ± 1.5

Pharmacological parameters are from non linear regression; pD_2 _is the negative logarithm of the concentration producing 50% of the maximum effect, EC_50 _is the concentration producing 50% of maximum effect (95% confidence limits are given in parenthesis), E_max _is the maximum effect (vasoconstriction to PE or ADMA in % of vasoconstriction evoked in the same preparations by 100 mmol/L K^+^; vasodilatation to ACh or SNP as % of residual tone). Statistical comparisons were made on the whole curves by two-way ANOVA; n = 12 preparations from 6 mice in each group; *P < 0.05, **P < 0.01 versus db/m + vehicle, ^††^P < 0.01 versus db/db + vehicle.

## Discussion

Endothelial dysfunction plays a pivot role in the initiation of the cascade of events leading to CVD [3]. ADMA is an eNOS inhibitor whose levels rise in a number of pathological conditions associated with endothelial dysfunction, including diabetes, dyslipidemia and obesity [9,10]. In type 2 diabetes, ADMA levels are associated with macrovascular diseases independently of traditional cardiovascular risk factors [15] and ADMA pathway has been proposed as a link between inflammation and endothelial dysfunction [27].

In this study, we report, for the first time, the association of ADMA levels with endothelial dysfunction in db/db mice and demonstrated that silibinin, a worldwide-used flavolignan from milk thistle, favorably impacts on ADMA levels and improves endothelial dysfunction. We found that db/db mice exhibit, both in plasma and aorta, ADMA levels higher than db/m mice. In silibinin-treated db/db, the substantial reduction of plasma and vascular ADMA is associated with the improvement of insulin resistance; this is in accordance with data in humans indicating that serum ADMA predicts the degree of insulin sensitivity [16]. Interestingly, circulating ADMA is not related to brachial artery-flow mediated dilatation in patients with type 1 diabetes [28]. The pathways involved in the beneficial effect of silibinin on insulin resistance are not known; however, a possible explanation for the decrease in blood glucose levels in treated animals may be the inhibition of liver gluconeogenesis [29]. The molecular link between insulin signaling and ADMA homeostasis is poorly understood at present; data obtained in endothelial cells suggest that insulin and adiponectin modulate the activity of DDAH [30]. Of note, DDAH gene variants influence the risk of diabetes and its complications [31].

Vascular endothelium is not the only contributor to regulation of plasma ADMA levels [12], and the role of other tissues and organs in ADMA metabolism remains to be elucidated. Interestingly, in this study we found not only a positive correlation between plasma and vascular ADMA, but also an inverse correlation between liver and both plasma and aorta ADMA. The molecular basis for the observed correlations is not clear; we can speculate that the increase of hepatic ADMA in db/db mice induced by silibinin may involve the system of y^+ ^carriers of the cationic amino acid family, which has been shown to play a relevant role in the clearance of dimethylarginines in the liver [32]. In this respect, further studies in mice with diet-induced obesity/diabetes might be helpful. Similarly, the role of kidney in ADMA clearance should also be elucidated, although previous findings, showing that ADMA clearance is conserved in nephrectomized mice [33], indicate that it may be less substantial.

Our observation of impaired endothelium-dependent vasodilatation to ACh in db/db mice is consistent with previous reports [19-24]. Moreover, we found that endothelium-dependent vasodilatation of isolated aorta to ACh was improved by silibinin in db/db mice; because silibinin reduced ADMA levels in aorta, the subsequent reduction of NOS inhibition by ADMA may, at least in part, account for the observed improvement of endothelium-dependent vasodilatation. Surprisingly, despite the effect on aorta ADMA levels, silibinin did not modify *in vitro *vascular responsiveness to either PE or high K^+^. Enhanced vasoconstriction to α-adrenergic stimulation has been attributed to reduced basal nitric oxide production in db/db mice [19]; since silibinin did not change the concentration-response curve to PE, additional mechanisms, other than increase in ADMA levels, must be postulated for the reduced basal NO production in db/db mice; among them, free radical generation has been documented and may play an important role. Increased generation of oxygen free radicals is supposed to increase NO scavenging (with production of reactive species such as peroxynitrite), thereby reducing the vasodilator effectiveness of endogenous or exogenous (from NO-donor drugs) NO. In accordance with this hypothesis, endothelium-independent vasodilatation to SNP has also been shown to be reduced in db/db mice [23]. Here we confirm this latter issue, showing a slight, but significant, displacement to the right of the concentration-response curve to SNP in db/db mice. Because SNP is a NO-donor, reduced sensitivity to SNP implies reduced sensitivity to NO and/or increased NO scavenging in db/db vessels. Of note, the reduced responsiveness to SNP in db/db mice was not changed by silibinin treatment. Such a reduced sensitivity to NO in db/db vessels may also, at least in part, account for the reduced vasodilatation to ACh; however, the fact that silibinin improved the response to ACh, while not changing the response to SNP, would indicate that in silibinin-treated animals ACh-induced NO production was increased, possibly because endogenous ADMA was reduced, leading to increased NOS activity. Finally, when applied *in vitro*, ADMA elicited the same contractile response in the two groups of db/db mice, regardless of the treatment, suggesting that silibinin *in vivo *may inhibit ADMA production rather than enhance its vascular degradation.

## Conclusions

In conclusion, in this study we showed that plasma ADMA levels are increased in db/db animals similarly to diabetic patients. The increased ADMA levels are associated to endothelial dysfunction in db/db. Silibinin treatment reversed plasma and aorta ADMA increase and improved both endothelial dysfunction and insulin resistance. These pharmacological properties of silibinin are of potential clinical interest given its favorable safety profile.

## List of abbreviations

CVD: cardiovascular diseases; NO: nitric oxide; ADMA: asymmetric N^G^:N^G^-dimethyl-L-arginine; NOS: nitric oxide synthase; eNOS: endothelial nitric oxide synthase; DDAH: dimethylarginine hydrolase; HOMA: homeostasis model of assessment; IR: insulin resistance; PE: phenilephrine; Ach: acetylcholine; SNP: sodium nitroprusside.

## Competing interests

The authors declare that they have no competing interests.

## Authors' contributions

GLV conceived and designed the study, performed experiments, analyzed data and contributed to the writing of the manuscript; SS designed the study, performed experiments and contributed to the writing of the manuscript; VS performed experiments, analyzed data and critically reviewed the manuscript; AM analyzed data and critically reviewed the manuscript; VU performed experiments and analyzed data; IS performed experiments and analyzed data; FG analyzed data and critically reviewed the manuscript; FS conceived and designed the study, performed experiments, analyzed data and contributed to the writing of the manuscript.

All authors read and approved the final manuscript.
